# A review of the roles of pathogens in Alzheimer’s disease

**DOI:** 10.3389/fnins.2024.1439055

**Published:** 2024-08-19

**Authors:** Meng Zhao, Yongchun Wang, Yanxin Shen, Chunxiao Wei, Guimei Zhang, Li Sun

**Affiliations:** Department of Neurology, Neuroscience Center, The First Hospital of Jilin University, Jilin University, Changchun, China

**Keywords:** Alzheimer’s disease, bacterial infection, fungal infection, pathogen, viral infection

## Abstract

Alzheimer’s disease (AD) is one of the leading causes of dementia and is characterized by memory loss, mental and behavioral abnormalities, and impaired ability to perform daily activities. Even as a global disease that threatens human health, effective treatments to slow the progression of AD have not been found, despite intensive research and significant investment. In recent years, the role of infections in the etiology of AD has sparked intense debate. Pathogens invade the central nervous system through a damaged blood–brain barrier or nerve trunk and disrupt the neuronal structure and function as well as homeostasis of the brain microenvironment through a series of molecular biological events. In this review, we summarize the various pathogens involved in AD pathology, discuss potential interactions between pathogens and AD, and provide an overview of the promising future of anti-pathogenic therapies for AD.

## 1 Introduction

Alzheimer’s disease (AD) is a common neurodegenerative disease that has gained worldwide attention in recent years. Its main clinical manifestations include memory loss, mental and behavioral abnormalities, and impaired ability to perform daily activities. The situation is grim for the aging population as the incidence of AD is increasing annually around the world, imposing a huge economic burden. Without effective treatments, the number of people with AD will reach 150 billion by 2050, and its economic cost will reach $9.12 trillion (Jia et al., 2018; Alzheimers Dement., 2021). These data prompted us to increase our research on AD.

AD is a multifactorial disease with several proposed causes. Genetic factors, such as mutations in the APP, PSEN1, and PSEN2 genes and the presence of the APOE gene type 4 allele (APOE-ε4), are strongly associated with an increased risk of AD (Hardy and Selkoe, 2002; Cacace et al., 2016). Environmental factors, including exposure to toxins and traumatic brain injuries, and lifestyle factors, such as diet and physical activity, contribute to the risk of developing AD (Plassman et al., 2000; De la Rosa et al., 2020). The main pathological features of AD include senile plaques formed by amyloid-β (Aβ) aggregation and nerve fiber tangles composed of hyperphosphorylated tau proteins. Others features are defective mitochondria, oxidative stress, destabilized metal ion metabolism, over-activated glial responses, and neuroinflammation, leading to synaptic dysfunction, neuronal atrophy, and cognitive impairment (Yan et al., 2013).

The idea that infectious agents in the brain are involved in the pathogenesis of AD was proposed nearly 30 years ago, but this theory had failed to gain substantial traction (Ecarnot et al., 2023). In recent years, new evidence has emerged showing that various pathogens disrupt the brain microenvironment, trigger inflammatory cascade responses, and contribute to AD, suggesting an important nexus between pathogens and AD. For example, infections caused by herpes simplex virus type 1 (HSV-1), Chlamydia pneumoniae, and *Porphyromonas gingivalis* have been implicated in AD pathology (Wozniak et al., 2009; Ma et al., 2022). These pathogens can invade the central nervous system (CNS) through a damaged blood-brain barrier (BBB) or other neural pathways, disrupting neuronal structure and function through cascading molecular biological events (Wang et al., 2004; Wohlfert et al., 2017; Dominy et al., 2019). This review provides a comprehensive summary of various pathogens involved in AD pathology, discusses their potential interactions with AD, and outlines the promising future of anti-pathogenic therapies for AD.

## 2 Pathogens drive AD pathology

**FIGURE 1:**
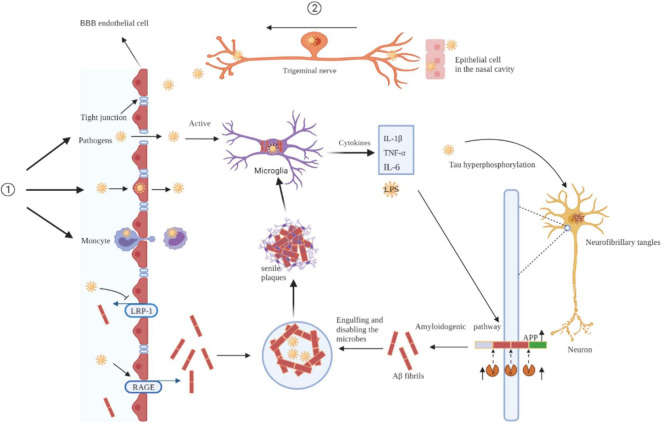
Mechanisms of pathogens invade the brain and pathogens drive AD pathology. Pathogens invade the CNS through a damaged BBB (①) or nerve trunk (②). Pathogens lead to sustained microglial activation and release of large amounts of inflammatory cytokines, ultimately leading to a chronic neuroinflammatory environment. Additionally, aggregation of Aβ and hyperphosphorylated tau proteins interact with pathogens to exacerbate the pathological events of AD. BBB, blood-brain barrier; IL-1β, interleukin-1β; TNF-α, tumor necrosis factor-α; LRP-1, low-density lipoprotein receptor-ligated protein-1; RAGE, receptor for advanced glycation end products; APP, amyloid precursor protein.

Mounting evidence suggests that pathogens are a major factor in neurodegenerative processes and may be upstream contributors to the pathological changes in AD. Pathogens invade the CNS through a damaged BBB or nerve trunk and disrupt neuronal structure, function, and homeostasis of the brain microenvironment through a series of molecular and cellular events. Pathogens lead to sustained microglial activation and release of large amounts of inflammatory cytokines, ultimately leading to a chronic neuroinflammatory environment. Additionally, aggregation of Aβ and hyperphosphorylated tau proteins, two key hallmarks of AD, interact with pathogens to exacerbate the pathological events of AD (Figure 1).

### 2.1 Initial infection and pathogen invasion

Pathogens can enter a host through a damaged barrier. Once they enter a host, they guarantee their proliferation and dissemination with the help of molecular mimetic mechanisms, which interfere with the host’s immune response and can eventually lead to a pathological response cascade. How do pathogens access the CNS (Figure 1)? We summarizes both human and rodent data to provide a comprehensive overview of the mechanisms involved.

#### 2.1.1 BBB disruption

The BBB comprises endothelial cells with several tight junctions that restrict the entry of blood-derived molecules and pathogens into the CNS (Wohlfert et al., 2017). Pathogens such as bacteria, viruses, fungi, and protozoa invade the CNS through BBB disruption and nerve trunk pathways. Once inside the brain, these pathogens trigger the release of inflammatory mediators and activate the host’s innate immune responses.

Viral infections disrupt the BBB through various mechanisms. For instance, HSV-1 can induce the release of pro-inflammatory cytokines such as tumor necrosis factor (TNF), interferon (IFN)-β, IFN-γ, and IFN-λ, which weaken tight junctions and increase BBB permeability (Wang et al., 2004). The Zika virus E protein modulates the functions of human brain microvascular endothelial cells, leading to BBB breakdown and allowing viral particles to invade the central CNS (Kaur et al., 2023). Inflammatory responses triggered by viral infections can also recruit immune cells to the site, releasing matrix metalloproteinases that degrade extracellular matrix components and disrupt the structural integrity of the BBB (Persidsky et al., 2006).

Bacterial infections can similarly lead to BBB disruption. Streptococcus pneumoniae, a common cause of bacterial meningitis, can cross the BBB by directly infecting capillary endothelial cells and spreading through endothelial cell junctions (Jimenez-Munguia et al., 2021). In mice infected with Streptococcus pneumoniae, the bacteria can cross the BBB and induce meningitis through the induction of nitric oxide synthase-2 by interferon-γ, contributing to BBB dysfunction (Yau et al., 2016). Streptococcus pneumoniae can invade the brain by binding to pneumococcal adhesins RrgA and PspC, which interact with pIgR and PECAM-1 on endothelial cells, facilitating BBB penetration and meningitis development (Iovino et al., 2017). This process is often facilitated by the release of bacterial toxins and inflammatory cytokines that weaken the BBB. Neisseria meningitidis, another meningitis-causing pathogen, interacts with brain endothelial cells via type IV pili, leading to the formation of microcolonies and activation of intracellular signaling pathways that result in the opening of intercellular junctions, further promoting BBB penetration (Delbaz et al., 2020). The release of lipopolysaccharides (LPS) from gram-negative bacteria can trigger a strong inflammatory response, leading to the degradation of tight junction proteins and increased BBB permeability by inducing neuroinflammatory reactions (Delbaz et al., 2020).

Viral and bacterial infections can utilize the “Trojan horse” pathway, whereby infected lymphocytes or monocytes/macrophages cross the BBB, carrying pathogens into the CNS (McGavern and Kang, 2011). This mechanism is particularly notable in chronic infections, where continuous low-level pathogen presence can lead to sustained inflammation and progressive BBB disruption. Studies have demonstrated that infections can lead to significant BBB disruption, allowing pathogens to enter the brain.

#### 2.1.2 Nerve trunk pathway

Pathogens may infect the epithelial cells of the oral or nasal mucosa and reach the trigeminal ganglion via olfactory bulb conduction. Certain viruses can evade immune system detection and hide in a latent state within the ganglion (Sait et al., 2021). If the host becomes immunocompromised, the latent virus is reactivated and projected by the trigeminal ganglion neurons to the trigeminal nucleus in the brainstem, followed by the thalamus, and finally to the sensory cortex (Piacentini et al., 2014).

Chlamydia pneumoniae can enter the nasal cavity, penetrate the olfactory epithelium, travel to the olfactory bulb, and subsequently, enter the olfactory cortex and hippocampus, leading to the formation of Aβ plaques and intracellular neurofibrillary tangles (NFTs) (Sundar et al., 2020). Studies have supported this mechanism, where similar pathways have been implicated in the spread of herpes simplex virus (HSV) to the CNS.

The BBB disruption pathway is supported by substantial data on humans and animals, suggesting it is a well-established route for pathogen invasion. The nerve trunk pathway appears to be more specific to certain types of pathogens, such as viruses that can remain latent in neuronal ganglia. The choice of mechanism may depend on the pathogen involved and the specific circumstances of the infection.

#### 2.1.3 Pathogen spread in the CNS

Once pathogens enter the CNS, they utilize various mechanisms to spread within the brain. Firstly, pathogens employ molecular mimicry to interfere with the host’s immune response, ensuring their proliferation and dissemination (Readhead et al., 2018). By imitating the host’s molecular structures, pathogens can evade immune system recognition and clearance, allowing them to persist and continuously trigger pathological responses (Oldstone, 1998; Friedland, 2015). Additionally, epitope spreading, a process where the immune response initially targets specific pathogen antigens and subsequently spreads to target the host’s own tissues, contributes to the persistence and dissemination of pathogens within the CNS (Readhead et al., 2018).

Some pathogens maintain low-level chronic infections within the host, leading to sustained inflammation and progressive BBB disruption. For instance, *Toxoplasma gondii* infection can cause neuroinflammation and BBB disruption, affecting the behavior of mice (Castano Barrios et al., 2021). Systemic chronic inflammation, such as that caused by the gut microbiota, is associated with neuroinflammation and neurodegeneration, further impacting the integrity of the BBB (Mou et al., 2022). HSV-1 is a well-known pathogen that can reactivate during periods of host immunosuppression, leading to cumulative neural damage and the formation of Aβ plaques associated with AD (Xie et al., 2021).

Once inside the CNS, pathogens activate microglia and astrocytes, resulting in persistent neuroinflammation. While this response helps clear pathogens, prolonged inflammation leads to neuronal damage and accelerate AD progression (Al-Ghraiybah et al., 2022). The continuous activation of these glial cells produces inflammatory cytokines and oxidative stress, exacerbating neuroinflammation and neuronal damage, thereby accelerating the pathological progression of AD (Raine, 2000). Microglia and astrocytes play crucial roles in this neuroinflammatory response, which is key in neurodegenerative diseases (Heneka et al., 2015).

### 2.2 Pathogen-induced inflammation and microglial activation in AD

Microglia are the resident phagocytes of the CNS and the first line of defense against pathogens invading the brain (Wang et al., 2018). When pathogens breach the CNS, they trigger the release of inflammatory mediators, initiating an innate immune response. Under normal physiological conditions, this acute inflammatory response aids in damage repair and the restoration of brain homeostasis. However, in the presence of chronic inflammatory triggers, microglia can become persistently activated, transforming into pro-inflammatory subtypes. This transformation leads to increased production of cytokines such as IL-1β, IL-6, and TNF-α, and an upsurge in reactive oxygen species (ROS) and reactive nitrogen species (RNS) (Sansores-España et al., 2021; Wainberg et al., 2021). These reactive molecules contribute to oxidative damage, promoting the progression of neurodegenerative processes. Oxidative stress further exacerbates the inflammatory response, resulting in uncontrolled inflammation, widespread cytokine circulation, neuronal damage, apoptosis, and the progression of chronic diseases (Lopatko Lindman et al., 2019; Qin and Li, 2019). Additionally, interactions between microglia and astrocytes enhance the secretion of pro-inflammatory cytokines, and the production of amyloid precursor protein (APP), and β- and γ-secretases, ultimately leading to the deposition of Aβ (Lin et al., 1995; Sansores-España et al., 2021).

Moreover, pathogenic bacteria and their components, such as lipopolysaccharides (LPS)—a potent endotoxin—are involved in persistent inflammation and Aβ accumulation in AD (Miklossy, 2008). Experimental models have shown that LPS-induced inflammation in APP transgenic mice not only increases APP expression but also stimulates β-secretase (BACE) activity, directly influencing Aβ production (Foyn Bruun et al., 1994; Wozniak et al., 2011). This demonstrates that pathogens and their derivatives are significant contributors to the inflammatory and amyloid pathology in AD.

### 2.3 Pathogens interact with Aβ in AD

#### 2.3.1 Aβ as an antimicrobial peptide

Traditionally, Aβ is considered an abnormal byproduct of APP metabolism. Excessive Aβ deposition plays a detrimental role in the pathology of AD. Therefore, most studies have aimed to treat AD by eliminating Aβ. However, these treatments are not only ineffective but also increase the incidence of infection. This observation has been reported in several relevant clinical trials. A clinical trial of tarenflurbil, which selectively lowers Aβ_42_, found a significant rise in infection rates in patients who received the drug (Green et al., 2009). These findings suggest that Aβ might be a potential antimicrobial peptide (AMP) that plays an important anti-infective role. Recent studies have found that Aβ has anti-infection activities (Vigasova et al., 2021), which inhibits the replication of the influenza A virus (IAV) *in vitro* (White et al., 2014). Additionally, increasing evidence suggests that Aβ functions as a natural antimicrobial barrier *in vivo*. One study demonstrated that the antimicrobial activity of Aβ peptides was equivalent to or greater than that of known antimicrobial peptide LL-37 against seven pathogens (Soscia et al., 2010). Aβ peptides can self-assemble into oligomers and aggregate into fibrillar networks that target, trap, and agglutinate microbes (Iqbal et al., 2020).

Furthermore, Aβ is believed to play a role in defending against bacteria, fungi, and viruses, providing more support for the infection hypothesis in explaining Aβ’s role in AD (Fulop et al., 2018). These findings strongly support the antimicrobial function of Aβ, redefining our understanding of Aβ’s role in AD.

#### 2.3.2 Antimicrobial mechanism of Aβ

Aβ microbe agglutination is mediated through the heparin-binding domain, which targets carbohydrates on the surface of pathogens. After entrapment, Aβ induces cell membrane disruption by forming cation channels and enhances the phagocytosis of entrapped pathogens by neutrophils and macrophages (Iqbal et al., 2020). This antimicrobial mechanism underscores the complex role of Aβ in maintaining CNS health while also posing a risk for AD development through chronic inflammation (Jorfi et al., 2023). Chronic neuroinflammation triggered by persistent infections can lead to continuous production and deposition of Aβ, forming plaques that are characteristic of AD (Jorfi et al., 2023). This inflammatory response involves the innate immune system and affects neuronal health and function. Neutrophils and their granule proteins play a significant role in the neuroinflammatory process by interacting with Aβ and contributing to its deposition (Stock et al., 2018). Moreover, studies have shown that Aβ can bind to specific cellular receptors, such as receptor for advanced glycation end-products (RAGE), which further amplifies the inflammatory response and contributes to the pathogenesis of AD (Stock et al., 2018). This highlights the need for therapeutic strategies that can modulate Aβ’s antimicrobial functions without exacerbating AD pathology.

#### 2.3.3 Pathogen invasion and Aβ production

Under normal conditions, APP metabolites maintain a balance between Aβ generation and elimination through a self-regulatory mechanism. Aβ clearance may involve clearance from the brain into the peripheral blood or degradation in the CNS tissues (Zuroff et al., 2017). In addition to being produced in the brain, Aβ may be produced by macrophages in the peripheral organs and spread to the brain (Nie et al., 2019; Olsen and Singhrao, 2020). Low-density lipoprotein receptor-ligated protein (LRP)-1 is a large multifunctional scavenger and signaling receptor expressed in the brain capillary endothelium (Herz and Strickland, 2001) and is a major transporter in Aβ efflux from the BBB. Aβ can be transferred from the brain to the peripheral blood via LRP on the surface of BBB cells (Deane et al., 2008). Conversely, the receptor for advanced glycation end products (RAGE), a multiligand receptor in the immunoglobulin superfamily, mediates the transport of circulation Aβ across the BBB to the brain (Deane et al., 2003, 2008). Pathogens upregulated the expression of RAGE by activating Toll-like receptor 2/nuclear factor κB (TLR2/NF-κB) signaling, which promotes Aβ entry into the brain (Nie et al., 2019). This pathogen-induced upregulation highlights the intricate relationship between infection, inflammation, and Aβ dynamics in AD.

### 2.4 Pathogens interact with tau in AD

#### 2.4.1 Bacterial interactions with tau protein

Bacterial infections can exacerbate tau pathology through several mechanisms. Bacterial components like LPS are known to induce neuroinflammation, leading to the activation of kinases that phosphorylate tau protein. This phosphorylation promotes tau aggregation, a hallmark of AD (Sy et al., 2011).

Abnormal phosphorylation of tau proteins disrupts neuronal structures and synaptic plasticity, leading to neuronal dysfunction and death (De-Paula et al., 2012). For instance, Wang X. et al. (2015) demonstrated that *H. pylori* induced significant hyperphosphorylation of tau proteins in mouse neuroblastoma (N2A) cells and glycogen synthase kinase 3β (GSK-3β)-activated rat brains at certain sites, including Thr205, Thr231, and Ser404. They found that GSK-3β inhibitors effectively reduced *H. pylori*-induced tau hyperphosphorylation (Wang X. et al., 2015). Moreover, Miklossy (2011) provided evidence that spirochetes in the brain can directly interact with tau, promoting its abnormal modification and aggregation. Additionally, gingipains, produced by *Porphyromonas gingivalis*, are found in the brains of AD patients, and their levels are closely related to tau and ubiquitin pathology (Dominy et al., 2019). Gingipains can activate caspase-3 (Urnowey et al., 2006) and aggravate tau phosphorylation (Chu et al., 2017; Dominy et al., 2019), further damaging neuronal function. Collectively, these results indicate that pathogens play an important role in tau phosphorylation.

#### 2.4.2 Viral interactions with tau protein

Viral infections also play a significant role in tau pathology through various pathways. Viruses can disrupt cellular processes and induce inflammatory responses that exacerbate tau pathology. Brunello et al. (2020) highlighted that HSV-1 can increase tau phosphorylation and aggregation by disrupting cellular processes and activating inflammatory pathways. Some viral proteins can interact directly with tau or tau kinases, modifying tau protein and promoting its aggregation, suggesting a direct mechanism through which viruses contribute to AD pathology.

#### 2.4.3 Immune response and tau pathology

The immune system’s response to pathogens can indirectly affect tau pathology. Microglia, the brain’s resident immune cells, become activated in response to pathogens and release cytokines and other inflammatory mediators that can lead to tau hyperphosphorylation (Jiang et al., 2018). Perea et al. (2020) discussed how microglial activation in the presence of pathogens contributes to the progression of tau pathology in AD. The release of inflammatory mediators by microglia and other immune cells can activate tau kinases, resulting in tau phosphorylation and aggregation. This highlights the indirect pathway through which pathogens contribute to tau pathology.

In summary, pathogens can activate a series of inflammatory responses, leading to the release of cytokines and other inflammatory mediators, which not only disrupt neuronal function but also promote the aggregation of Aβ and hyperphosphorylated tau proteins, the hallmark features of AD. Recent studies have shown that although Aβ and tau induce changes in neuronal plasticity, connections, and activities in similar ways, they do so through different mechanisms and pathways (Busche et al., 2012; Frandemiche et al., 2014; Yang et al., 2018; Lin et al., 2022). Both Aβ and tau contribute to the disruption of neuronal function in AD, and their interactions with pathogens further exacerbate these pathological events.

## 3 Contribution of pathogens to AD pathogenesis

### 3.1 Viruses contributions to AD

#### 3.1.1 COVID-19 and Alzheimer’s disease interactions

Since the onset of the COVID-19 pandemic, emerging evidence has suggested that the SARS-CoV-2 virus could have lasting impacts on the CNS, potentially exacerbating neurodegenerative diseases such as AD (Krishna et al., 2023). The virus’s neuroinvasive capabilities and its potential to accelerate the neuropathological processes underlying AD draw particular attention (Wang et al., 2022).

##### 3.1.1.1 Systemic inflammation and its impact on AD

The systemic inflammation triggered by COVID-19 has been noted for its potential to exacerbate the chronic neuroinflammation already present in AD (Griggs et al., 2022; Ortiz et al., 2022). Elevated inflammatory cytokines, such as IL-6 and TNF-alpha, in both COVID-19 patients and individuals with AD suggest a common pathway of immune response exacerbation (Tsagkaris et al., 2022; Krishna et al., 2023). This heightened inflammatory state can accelerate the deposition of amyloid-beta in the brain, a hallmark of AD pathology (Chen et al., 2023). Furthermore, the pro-inflammatory cytokines can activate microglia, the brain’s resident immune cells, which may in turn increase the production of amyloid-beta and promote the formation of neurofibrillary tangles, thereby speeding up the cognitive decline observed in AD (Piekut et al., 2022; Chen et al., 2023).

##### 3.1.1.2 Blood-brain barrier disruption

COVID-19 has been associated with increased permeability of the BBB, a critical defense mechanism that limits the entry of pathogens and inflammatory mediators into the CNS (Chen et al., 2022). Disruption of the BBB by SARS-CoV-2 can facilitate the ingress of peripheral immune cells and cytokines into the brain, intensifying the neuroinflammatory response and potentially leading to an increase in neurodegenerative changes associated with AD (Chen et al., 2022; Griggs et al., 2022; Tsagkaris et al., 2022; Greene et al., 2024). This disruption might also alter the clearance mechanisms of amyloid-beta, contributing further to its accumulation in the brain (Zenaro et al., 2017).

##### 3.1.1.3 Direct neuronal damage and AD

Beyond the indirect effects through systemic inflammation and BBB disruption, COVID-19 might also cause direct neuronal damage due to the neurotropic nature of SARS-CoV-2 (Zhou et al., 2020). The virus has been detected in neural tissues and cerebrospinal fluid of infected individuals, suggesting that it can directly infect neurons and other brain cells (Zhou et al., 2020; Liu et al., 2023). This direct interaction could lead to cell death or functional impairment of neurons, particularly in regions of the brain critical for memory and cognition, such as the hippocampus and cortex, which are also significantly affected in AD (Liu et al., 2023).

##### 3.1.1.4 Long-term cognitive effects and research needs

The long-term cognitive sequelae of COVID-19 are still under investigation, with emerging studies indicating that even mild COVID-19 cases can result in significant cognitive disturbances that might last beyond the acute phase of the infection (Crivelli et al., 2022). For individuals with AD, or those at risk, COVID-19 could act as an accelerant, magnifying the disease’s progression and possibly precipitating an earlier onset of symptoms (Joo et al., 2022). Ongoing research is crucial to understand the full spectrum of COVID-19’s impact on AD, including potential genetic factors that might influence individual susceptibility to exacerbated AD progression in the context of COVID-19 (Griggs et al., 2022; Joo et al., 2022).

#### 3.1.2 HSV-1

##### 3.1.2.1 HSV-1 induced infections in AD

The idea that viruses play a role in the pathogenesis of AD was proposed 30 years ago by the discovery of HSV-1 DNA in the brains of elderly individuals in 1991 (Jamieson et al., 1991), followed by the evidence that HSV-1 antibodies were found in the cerebrospinal fluid (CSF) of most AD patients and normal controls (Wozniak et al., 2005). A strong link between AD and HSV was proposed soon after the discovery of the co-localization of HSV-1 DNA within amyloid plaques in AD and the recognition of an increased risk of AD in HSV-IgM seropositive individuals (Letenneur et al., 2008; Wozniak et al., 2009). As the primary virus causing AD, HSV-1 infects most people in infancy and then remains dormant in the peripheral nervous system (PNS). Occasionally, if a person is stressed, the virus is activated, and in some people, it causes cold sores (Harris and Harris, 2018). As for the elderly, it is thought that in most countries, 80–90% of people are infected with HSV1 by the age of 60 years (Itzhaki, 2016).

The decline of the immune system and allows HSV-1 to enter the CNS from the PNS. It can also be reactivated, perhaps repeatedly, during events such as immunosuppression, causing inflammatory processes and direct viral actions (Itzhaki, 2016; Itzhaki and Lathe, 2018). CNS inflammation, although it may be very localized, is a type of “mild” encephalitis, leading to consequent neuronal damage (Itzhaki, 2016). It is important to emphasize that herpes encephalitis affects the same regions of the brain as those in AD (hippocampus, and frontal cortical areas) (Itzhaki et al., 2016). Repeated reactivation leads to accumulated neuronal damage and the formation of amyloid plaques and NFTs. HSV-1 infection can cause significant Aβ deposition and AD-like tau phosphorylation in infected cell cultures and animal models. For instance, intracerebral injection of HSV-1 in 5xFAD transgenic mice resulted in the accumulation of Aβ plaques within 48 h (Wozniak et al., 2007; Eimer et al., 2018). The rapid spread of viral particles triggers host immune responses, leading to Aβ accumulation as an antimicrobial response. Subsequent immune activation and inflammatory cascades result in large Aβ deposits and uncontrolled inflammation, ultimately contributing to AD pathology (Kumar et al., 2016; Eimer et al., 2018). These findings highlight the importance of the temporal dynamics of HSV-1 infection and its effects on Aβ deposition (Itzhaki, 2017; De Chiara et al., 2019; Li Puma et al., 2019; Wainberg et al., 2021).

##### 3.1.2.2 HSV-1 interacts with APOE in AD

Mounting evidence shows that HSV is a strong contributory risk factor for AD, significantly increasing the risk of AD when present in the brains of carriers of the APOE-ε4, neither of which by itself poses a substantial risk (Itzhaki, 2017; Lovheim et al., 2019; Khokale et al., 2020). Statistically, 60% of patients with AD who carry the virus in the brain are also APOE-ε4 carriers (Itzhaki, 2016). The interaction of APOE-ε4 heterozygosity (APOE-ε2/ε4 or -ε3/ε4) and HSV-1 carriage increases AD risk by approximately 12-fold, whereas the presence of only one factor poses a much lower risk (Khokale et al., 2020). However, studies in 5xFAD transgenic mice have demonstrated that HSV-1 infection can independently induce AD-like pathology, regardless of the APOE genotype (Eimer et al., 2018). This suggests that while APOE-ε4 may exacerbate HSV-1-induced neurodegeneration, HSV-1 alone can trigger significant Aβ and tau pathology. Therefore, genetic and viral factors are crucial in AD pathogenesis, and their interactions warrant further investigation.

It is believed that APOE-ε4 is one of the important molecules that regulate the immune system and that it can control the infection outcome from several pathogens. First, it was found that APOE-ε4 is a risk factor for cold sores (herpes labialis) (Lin et al., 1995; Itzhaki et al., 1997), a disease caused by HSV-1. Second, another variant of APOE (APOE-ε2) is a risk factor for severe HSV-1 encephalopathy (Lin et al., 2001). Therefore, APOE plays a crucial role in determining the consequences of infection with the pathogen, strengthening the belief that HSV-1 and APOE-ε4 are an important pair of factors in AD.

Individuals with APOE-ε4 suffer greater viral damage or can repair less damage upon HSV reactivation and are therefore at risk of developing AD (Jamieson et al., 1991; van Exel et al., 2017; Lopatko Lindman et al., 2019). Several possible mechanisms have been proposed. First, APOE and HSV-1 may compete for cell entry through a common receptor, heparan sulfate proteoglycan (HSPG); thus, an APOE isoform that is poor at competing with HSV-1 will allow the entry of more viral particles into the cells, causing more damage (Lin et al., 2002). In the case of AD, APOE-ε4, compared to other isoforms, likely competes less adequately with HSV-1 for entry into neuronal cells. The second proposed explanation involves cellular repair after viral damage. After neuronal damage, APOE accumulates at the site of injury and participates in repair by removing excess lipids that may be neurotoxic and/or by supplying lipids necessary for repair. APOE isoforms differ in their ability to repair neuronal cells, leading to reduced repair capacity in some (Itzhaki and Wozniak, 2006). Thus, genetic susceptibility to HSV-1 reactivation and individual immune response to Aβ deposition induced by HSV-1 infection may be the direct mechanisms responsible for the pathological damage of AD. Based on the above, the use of antiviral drugs for APOE-ε4 carriers is warranted, given the prevalence of HSV-1 infection.

#### 3.1.3 Other viruses

In addition to the involvement of HSV-1 in AD, other members of the herpes virus family, namely human herpesvirus 6/7 (HHV-6/7), herpes simplex virus type 2 (HSV-2), and cytomegalovirus (CMV), have also been detected in the brains of patients with AD or have been associated with its pathogenesis (Piacentini et al., 2014). Other types of viruses, such as the hepatitis B virus (HBV), hepatitis C virus (HCV), and human immunodeficiency virus (HIV) are also associated with AD.

According to the previous study, HHV-6 and -7 are thought to have the highest loads in the brains of patients with AD (Readhead et al., 2018), although the authenticity of this finding has been questioned (Jeong and Liu, 2019). HHV-6 is much more prevalent in AD than in age-matched normal brains (70 vs. 40%) and overlaps extensively with HSV-1 in AD brains (Bourgade et al., 2015), but HHV-6, unlike HSV-1, is not directly associated with APOE-ε4 in AD (Itzhaki, 2016), and its pathogenic mechanism has not yet been elucidated. Some studies have shown that HHV-6 may be an environmental risk factor for cognitive decline and AD progression in elderly subjects. A 5-year study showed a 23% positivity rate for HHV-6 in the peripheral blood leukocyte samples from patients with AD, compared to 4% in controls (Carbone et al., 2014). HHV-7 is thought to be a co-effector of HHV-6. However, more research is required to gain further insights into the roles of HHV-6 and HHV-7 in AD. HSV-2, another herpes virus highly homologous to HSV-1, remains latent in sensory neurons but is capable of reactivation, and it can infect the brain and cause neurological symptoms, just like HSV-1 infection. HSV-2 infection has been found to cause increased accumulation of Aβ and hyperphosphorylated tau proteins, altered APP processing, and impaired autophagy (Kristen et al., 2015). In addition, a study in 2017 demonstrated that the incidence of dementia was 2.97 times higher in patients with herpes zoster ophthalmicus (HZO) due to HSV-2 than in the control group (Tsai et al., 2017). Cumulative evidence suggests that CMV is also involved in AD pathogenesis (Lovheim et al., 2018). Patients with anti-CMV seropositivity have a twofold increased risk of AD, faster cognitive decline, and more inflammatory markers (Westman et al., 2014; Barichello et al., 2015).

HCV infection significantly and independently increases the risk of dementia, and the severity of HCV infection is related to the degree of AD prognosis (Chiu et al., 2014). The pathology may be a direct viral infection or cognitive dysfunction caused by the infection of monocytes/macrophages, which subsequently secrete excessive amounts of cytokines that cause CNS excitotoxicity. Studies have also shown that liver dysfunction caused by HBV infection is possibly correlated with the level of Aβ in the plasma, suggesting that the liver may be involved in the peripheral clearance of Aβ. HBV infection and the related chronic inflammation may be involved in the pathogenesis of AD. Maintaining and restoring liver function and preventing HBV infection may prevent the development of AD (Wang et al., 2017).

Furthermore, HIV is associated with the presence of Aβ in the brain, and the amount of Aβ is related to the viral load, which is mainly found in neurons (Chakradhar, 2018). The postulated mechanism is that HIV activates the microglia and increases their APP expression. APP binds to the HIV-Gag polyprotein (the major structural protein of HIV-1) and retains HIV in lipid rafts, thereby preventing the production and spread of the virions. However, to evade the immune response, HIV-Gag promotes the secretase-dependent cleavage of APP, resulting in the overproduction of Aβ. Gag-mediated Aβ production leads to increased degeneration of the primary cortical neurons, which facilitates AD development (Chai et al., 2017).

### 3.2 Bacteria and AD

#### 3.2.1 Chlamydia pneumoniae

In Balin et al. (1998) were the first to report the existence of metabolically active *C. pneumoniae* in specific pathological regions of the brain in patients with AD. They detected *C. pneumoniae*-specific DNA and antigens in the brains of 90% (17 out of 19) of patients with AD and successfully cultured two strains of *C. pneumoniae* (Balin et al., 1998). Animal studies have shown that after intranasal infection with *C. pneumoniae* in non-transgenic BALB/c mice, Aβ deposits resembling plaques were found in the brains and *C. pneumoniae* was isolated from brains at 3 months of age (Little et al., 2004). *C. pneumoniae* is an obligate intracellular gram-negative bacterium that mainly infects the mucosal epithelium of the eyes, pulmonary, and urogenital systems (Cheok et al., 2020). If the local cellular environment is not suitable for replication, such as stressful environments with antibiotic cytokines or nutrient deficiency, the bacteria will enter a long-term, viable, but non-replicating state called chlamydia persistence. Once the stressor is eliminated, the *C. pneumoniae* can quickly return to normal replication (Panzetta et al., 2018). The persistent immune escape strategy in chronic chlamydial infection enhances the long-term survival of the pathogen and induces a chronic inflammatory state in the body (Wong et al., 2019). Upon entry into the brain, *C. pneumoniae* can infect various cells and remain in intracellular inclusion bodies that resist immune recognition and lysosomal fusion. Thereafter, *C. pneumoniae* promotes microglial secretion of pro-inflammatory cytokines such as IL-1β, IL-6, and TNF-α, which may directly increase Aβ production through the activation of BACE (Wyss-Coray, 2006). In addition, LPS secreted by *C. pneumoniae* can activate NF-κB signaling and promote the production of other inflammatory cytokines (Zhan et al., 2018).

#### 3.2.2 Spirochetes

Spirochetes are gram-negative, spiral-shaped, and the most neurophilic bacteria (Miklossy, 2015) that easily cross the BBB to invade the brain (de Vries et al., 1997). Spirochetes are associated with several chronic diseases, including syphilis (*Treponema pallidum*), Lyme disease (*Borrelia burgdorferi*), and gingivitis (*Treponema denticola*) (Miklossy, 2011). When the spirochetes enter the brain and slowly multiply in sufficient numbers, they create biofilms that activate the innate immune system (Allen, 2016). Biofilms protect microbes from toxic substances.

As early as 1910, Perusini analyzed the brains of four patients with AD and found that NFTs and plaques might be present in pre-existing cases of syphilitic dementia (Miklossy, 2015). *T. pallidum* is transmitted primarily through sexual contact and persists in the brain, leading to chronic inflammation and slow progression to dementia (Miklossy, 2015). *B. burgdorferi*, similar to *T. pallidum*, can also persist in host tissues and play a role in AD (Fallon and Nields, 1994). MacDonald and Miranda first cultured *B. burgdorferi* from the brains of patients with AD and proposed a possible link between AD and *B. burgdorferi* (MacDonald and Miranda, 1987). *B. burgdorferi* is the causative agent of Lyme disease transmitted to humans by the bite of an infected tick (Burgdorfer et al., 1982) and can induce the degradation of tight junction proteins between endothelial cells in the BBB and the brain (Grab et al., 2005). In *in vitro* studies, wherein mammalian glial and neuronal cells were exposed to *B. burgdorferi* and LPS, morphological changes analogous to the amyloid deposits in AD and hyperphosphorylation of tau protein were observed after 2–8 weeks (Miklossy et al., 2006). This result may be attributed to the induction of inflammation, leading to tau protein phosphorylation, microtubule dysfunction, and the generation of NFTs, which further leads to neurodegeneration. Lyme disease, caused by *B. burgdorferi*, and syphilis, caused by *T. pallidum*, lead to cortical atrophy and dementia in advanced stages (Honjo et al., 2009).

Animal studies have demonstrated that *T. denticola* can directly enter the brain and cause intracellular and extracellular accumulation of Aβ_1–40_ and Aβ_1–42_ in the hippocampi of C57BL/6 mice through selective activation of BACE and γ-secretase (Su et al., 2021). Both the BACE inhibitor KMI1303 and the γ-secretase inhibitor DAPT could reduce Aβ deposition (Su et al., 2021).

#### 3.2.3 *Porphyromonas gingivalis*

Periodontitis is a chronic inflammatory disease caused by *P. gingivalis* (Holt et al., 1988), *T. denticola*, *Tannerella forsythia* (Holt and Ebersole, 2000), and *Actinomyces* (Howard and Pilkington, 1992), which can damage the gums and surrounding tissues and influence a variety of diseases (Pihlstrom et al., 2005; Hajishengallis et al., 2012) such as AD (Qian et al., 2021) and Parkinson’s disease (Liu et al., 2013). A retrospective matched cohort study demonstrated that 10-year chronic periodontitis exposure was associated with a 1.707-fold increase in the risk of developing AD (Chen et al., 2017). It is reported that *P. gingivalis* can produce toxic substances such as LPS and gingipain, which play major pathogenic roles in the pathogenesis of periodontitis and AD. A study observed that after the injection of *P. gingivalis* LPS in the periodontal tissues, APP/PS1 mice showed learning and memory impairment, augmented Aβ and neuroinflammatory responses (Qian et al., 2021). Besides, a systematic review concluded that *P. gingivalis* infection or the use of *P. gingivalis* LPS increased the production of inflammatory mediators such as TNF-α, IL-6, and IL-1β, leading to inflammation and enhanced Aβ production (Costa et al., 2021). Infection with *P. gingivalis* can also upregulate the expression of RAGE, which can be regulated by NF-κB-dependent cathepsin B (CatB), and promote the entry of Aβ into the brain (Nie et al., 2019).

Furthermore, *P. gingivalis* can invade and survive in neurons and produce intraneuronal gingipains (Haditsch et al., 2020). Gingipains are cysteine proteases composed of lysine-gingipain (Kgp), arginine-gingipain A (RgpA), and arginine-gingipain B (RgpB), which have been identified in the brains of patients with AD, and their levels are correlated with tau and ubiquitin pathology (Dominy et al., 2019). Gingipains can activate caspase-3 (Urnowey et al., 2006) and cause tau phosphorylation (Chu et al., 2017; Dominy et al., 2019), leading to neuronal damage associated with AD.

#### 3.2.4 Helicobacter pylori

*H. pylori* is a spiral-like, gram-negative bacterium that spreads through the mouth and causes infection by attaching to the duodenum and is present in the gastrointestinal systems of more than 50% of adults (Malaguarnera et al., 2004). *H. pylori* can cause chronic gastritis, which, if left untreated, can lead to chronic inflammation (Albaret et al., 2020). Current studies have found that *H. pylori* infection can increase the risk of AD (Malaguarnera et al., 2004; Beydoun et al., 2018). Kountouras et al. (2009) demonstrated that *H. pylori*-specific IgG levels were significantly increased in the CSF and serum of patients with AD. Moreover, a research illustrated that *H. pylori* filtrates induce tau hyperphosphorylation at several AD-related tau phosphorylation sites in rat brains with GSK-3β activation, and application of GSK-3β inhibitors decreased *H. pylori*-induced tau hyperphosphorylation (Wang X. et al., 2015). These results indicate that *H. pylori* infection may be associated with abnormal tau hyperphosphorylation. Another study suggested that soluble surface fractions of *H. pylori* may promote Aβ_42_ formation by enhancing the activity of γ-secretase, thereby causing cognitive impairment by interrupting synaptic function (Wang et al., 2014).

#### 3.2.5 Gut microbiota (GM)

The GM is a diverse and dynamic population of microbes of approximately 100 trillion symbiotic microbial cells that reside in the GIT (Shabbir et al., 2021). The microbiota–gut–brain axis plays a key role in regulating brain function (Liu et al., 2021). A growing body of evidence suggests that intestinal flora dysregulation is related to the pathogenesis of AD; however, the specific mechanism is still unclear (Vogt et al., 2017). A study using 16S ribosomal RNA sequencing confirmed that there are significant differences in the composition of the GM between healthy individuals and patients with AD (Zhuang et al., 2018). Current research indicates that an increase in the abundance of pro-inflammatory GM taxa, such as *Escherichia* and *Shigella*, and a reduction in the abundance of anti-inflammatory taxa, such as *E. rectale*, is possibly associated with cognitive impairment and brain amyloidosis (Cattaneo et al., 2017). GM can affect the brain and immune system through the production of LPS and amyloid (Leblhuber et al., 2021). During aging, when both the GIT epithelium and BBB become more permeable, LPS and amyloids may pass directly through these protective physiological barriers or indirectly via LPS/amyloid-triggered cytokines, thereby leading to increased neuroinflammation and deposition of Aβ fibrils in the brain (Kesika et al., 2021).

In addition, alterations in GM composition lead to abnormally elevated levels of phenylalanine and isoleucine, stimulating the differentiation and proliferation of pro-inflammatory T helper 1 (Th1) cells (Wang et al., 2019). Peripheral Th1 cells then enter the brain and are associated with M1 microglial activation, which contributes to AD-related neuroinflammation (Wang et al., 2019). Recent studies have also shown that prebiotic mannan oligosaccharide (MOS) treatment significantly decreased the accumulation of Aβ and inhibited the expression of APP and BACE1 in the brains of AD mice (Wang et al., 2019; Liu et al., 2021). Therefore, restoring healthy GM through probiotic supplementation may provide a novel approach for the prevention and treatment of AD (Kesika et al., 2021).

#### 3.2.6 *Propionibacterium acnes*

*Propionibacterium acnes* is a slow-growing, rod-shaped, non-spore-forming, gram-positive, atypical anaerobe that colonizes numerous parts of the body, including the sebaceous follicles of the face and neck (Grice and Segre, 2011; Leheste et al., 2017). It is a relatively weak pathogen and tends to cause either self-limiting or chronic infections (Kornhuber, 1996), the most common disease being acne (Koreck et al., 2003). In Kornhuber (1996) was the first to find *P. acnes* in the cortex of three patients with AD. In this study involving nine brain tumors, *P. acnes* was found in 3 of 4 patients with AD and 1 of 5 control subjects (Kornhuber, 1996). *Propionibacterium acnes* can penetrate the brain through transcellular invasion of the BBB (Lu et al., 2018). At present, there are few studies related to the link between *P. acnes* and AD, which need to be investigated by further studies.

### 3.3 Fungi and protozoa in AD

Alonso et al. (2014) demonstrated the presence of fungal proteins and DNA in the brain tissues of patients with AD, using immunohistochemistry. Four years later, they demonstrated that the fungal genera more prevalent in patients with AD were *Alternaria*, *Botrytis*, *Candida*, and *Malassezia* using next-generation sequencing (NGS) (Alonso et al., 2018). This finding supports the association between fungi and AD.

At present, studies on the correlation between protozoa and AD have mainly focused on *Toxoplasma gondii*. *T. gondii* is an intracellular parasite, and the brain is the primary target organ (Li et al., 2019). Current studies have shown that *T. gondii* is associated with various neuropsychiatric disorders, such as AD (Kusbeci et al., 2011), Parkinson’s disease (Miman et al., 2010), and mental illnesses (Elsheikha et al., 2016). A study found higher anti-*T. gondii* IgG levels in patients with AD compared with control groups (Kusbeci et al., 2011). *T. gondii* enters the CNS through actin-myosin motors called the “gliding motility” pathway, in addition to the “Trojan horse” pathway (Ortiz-Guerrero et al., 2020). *T. gondii* infection not only induces the phosphorylation of tau by activating GSK-3β but also promotes the apoptosis of hippocampal neurons (Tao et al., 2020). Torres et al. (2018) showed that *T. gondii* infection induced Aβ immunoreactivity and hyperphosphorylated tau in the brains of C57BL/6 mice. However, Shin et al. (2021) showed that in the course of chronic infection, *T. gondii* induces the continuous proliferation of homeostasis-maintaining microglia *in vivo* and promotes apoptosis in cells after phagocytosis of Aβ plaques, without causing neuroinflammation. Chronic *T. gondii* infection may promote the clearance of Aβ plaques (Shin et al., 2021).

## 4 Targeting pathogens as therapeutic intervention for AD

**FIGURE 2:**
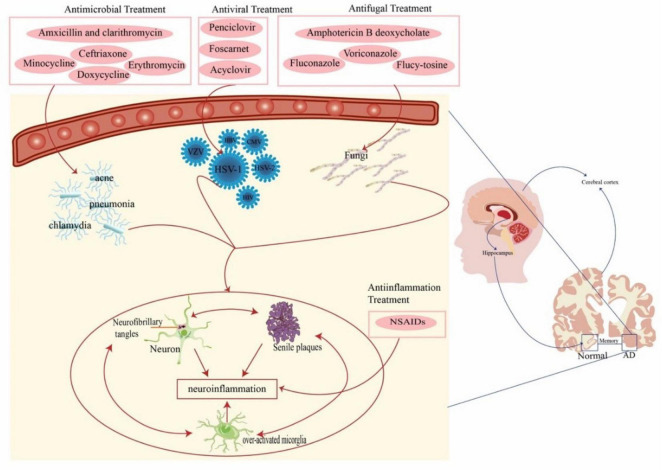
Anti-pathogenic/anti-inflammation therapies for AD. Pathogens (virus, bacteria, and fungi) act as risk factors for AD. Pathogens lead to sustained activation of microglia, induce aggregation of Aβ and hyperphosphorylated tau proteins, and release large amounts of inflammatory cytokines, ultimately leading to a chronic neuroinflammatory environment. This suggests that aggressive anti-infective therapies may be beneficial in slowing the progression of AD. AD, Alzheimer’s disease; HSV-1, herpes simplex virus type-1; HBV, hepatitis B virus; HCV, hepatitis C virus; HIV, human immunodeficiency virus; CMV, cytomegalovirus; NSAIDS, nonsteroidal anti-inflammatory drugs.

Currently, only four drugs are approved by the Food and Drug Administration (FDA) for the treatment of AD. Three of these are acetylcholinesterase inhibitors (donepezil, galantamine, and rivastigmine) and one is an N-methyl-D-aspartate (NMDA) receptor antagonist (memantine) (Alzheimers Dement., 2021). Additionally, anti-amyloid monoclonal antibody drugs Aducanumab and Lecanemab are currently used primarily for specific cases or are in clinical trial stages (Cummings et al., 2024). Despite intensive research and significant investment into understanding AD pathogenesis, none of these approaches have been shown to be effective in halting or slowing neurodegeneration in human clinical trials. The role of infectious agents in the etiology of AD has sparked intense debate in recent years, which suggests that pathogens may act as risk factors for AD and that aggressive anti-infective therapy can prevent or delay the progression of AD (Table 1 and Figure 2).

**TABLE 1:** Pathogen and inflammation-targeted therapies for AD.

Drug type	Major drugs	Efficacy in AD	Side effects	Limitations
Antiviral	Acyclovir, penciclovir, foscarnet	Reduces HSV-1 titer, Aβ accumulation, and tau phosphorylation	Gastrointestinal issues, headaches, renal toxicity	Long-term use may lead to antiviral resistance; efficacy in human AD patients requires further clinical validation
Antibacterial	Doxycycline, minocycline, erythromycin, rifampicin, amoxicillin, COR388	Reduces Aβ accumulation, tau phosphorylation, improves cognitive function, reduces pathological changes	Gastrointestinal disturbances, photosensitivity, potential impacts on microbiome, secondary infections	Long-term use may lead to antibiotic resistance and disruption of beneficial microbial flora; long-term safety and efficacy in humans require more research
Anti-inflammatory	Ibuprofen, aspirin, steroids	Reduces amyloid plaque deposition, tau phosphorylation, activation of astrocytes and microglia	Gastrointestinal issues, cardiovascular risks, renal impairment	Potential for serious side effects with prolonged use; inconsistent results in RCTs regarding efficacy in AD treatment

The table summarizes various treatments targeting pathogens in AD, highlighting their efficacy, side effects, and limitations. More research is needed to optimize these treatments and understand their long-term impacts on AD progression.

### 4.1 Antiviral treatment

Evidence suggests that some pathogens, especially viruses, play an important role in the pathogenesis of AD (Tzeng et al., 2018). Therefore, targeted antiviral therapy may be an important area to pursue in AD therapeutics. In several *in vitro* studies, anti-HSV-1 agents, including acyclovir, penciclovir, and foscarnet, have been analyzed and shown to reduce HSV-1 titer, Aβ accumulation, and tau phosphorylation (Wozniak et al., 2011). Two other studies investigated the possible relationship between dementia and the reactivation of another herpetic virus, varicella-zoster virus (VZV), and reported an increased risk of cognitive decline after HZO (Tsai et al., 2017) and a decreased incidence of dementia in HZO patients receiving anti-herpetic antiviral therapy.

Antiviral agents can downregulate the inflammatory response in the CNS and reduce the production of pro-inflammatory molecules, Aβ accumulation, and hyperphosphorylated tau proteins. Acyclovir, an antiviral agent, is a nucleoside analog that interferes with HSV-1 DNA replication by integrating into viral DNA to induce premature chain termination (Elion, 1982). It is primarily used for HSV infection, chickenpox, and shingles. After oral administration, valacyclovir is rapidly hydrolyzed to acyclovir in the intestines and liver and crosses the BBB to reach the CNS (Smith et al., 2010). In an *in vitro* study on HSV-1-infected kidney epithelial cells, acyclovir inhibited Aβ and phosphorylated tau accumulation and reduced HSV-1 protein levels in a concentration-dependent manner (Wozniak et al., 2011). The reduction in Aβ accumulation induced by antiviral therapy has been attributed to a reduction in viral replication (Panza et al., 2019). Penciclovir is a guanosine analog used to treat various herpesvirus infections. Foscarnet is a DNA polymerase inhibitor that is primarily used to treat *Herpesviridae* infections. Evidence has shown that both drugs can inhibit Aβ accumulation and tau protein phosphorylation induced by HSV-1, with foscarnet being less effective than penciclovir (Wozniak et al., 2011).

In summary, antiviral drugs can reduce the pathological damage in the AD brain. However, the long-term use of antiviral drugs may lead to antiviral resistance. Common side effects include gastrointestinal issues, headaches, and renal toxicity (Qin and Li, 2019). Although antiviral therapy has shown potential in animal models, its efficacy in human AD patients still requires further clinical validation. Whether antiviral drugs should be used in all patients with AD or only in those with confirmed viral infections remains to be further investigated to determine the optimal application strategy (Fulop et al., 2021).

### 4.2 Antibacterial treatment

Bacterial infections have been associated with the progression of AD. Previous studies have reported the presence of bacteria in the brain, suggesting the existence of a brain microbiome (Branton et al., 2016; Emery et al., 2017; Westfall et al., 2020). Although bacteria are also present in the brains of healthy individuals, tissue samples from AD brains have higher levels of bacterial (Emery et al., 2017), indicating higher levels of infiltration. Considering immunosuppression and other side effects, antibacterial therapy is certainly not suitable for long-term use, but infected patients are more likely to develop AD in the future; therefore, aggressive treatment is still recommended. The effects of tetracyclines, erythromycin, and rifampicin on AD, as well as advances in the treatment of *H. pylori* and *P. gingivalis* are briefly described below.

#### 4.2.1 Tetracyclines

Doxycycline is a tetracycline antibiotic that can exert direct effects in the CNS because it can penetrate the BBB (Balducci and Forloni, 2019). In *in vivo* studies, doxycycline was administered to mice, and its accumulation was observed in Aβ plaques (Merlini et al., 1995). Although doxycycline did not induce changes in the number of Aβ monomers and size of Aβ plaques in transgenic mice, Aβ 18-mer levels were significantly reduced compared to the control, and significant memory recovery was also observed in the treated animals (Balducci et al., 2018). This result may be attributed to the short study period of only two months, as another three-month study found a significant reduction in plaque size (Balducci et al., 2018). Longer clinical trials are needed to confirm these findings. Minocycline is another commonly used tetracycline. In the early stages of AD-like Aβ pathology, minocycline treatment (50 mg/kg for 4 weeks) attenuated behavioral abnormalities, neuroinflammatory markers, and Aβ accumulation in transgenic mouse models (Cuello et al., 2010). Based on its anti-inflammatory and neuroprotective properties, minocycline has been suggested as a treatment for AD patients with co-infectious pathologies (Budni et al., 2016). Erythromycin is a macrolide antibiotic used to treat several bacterial infections. A pilot study conducted in a TgCRND8 transgenic AD mouse model showed that treatment with erythromycin in drinking water (0.1 g/l) for 3 months resulted in 54% reduction in cortical Aβ_1–42_ levels compared to vehicle-treated animals (Tucker et al., 2005). These results were replicated in a further study on TgCRND8 mice (Tucker et al., 2006).

#### 4.2.2 Rifampicin

As a well-known drug for the treatment of tuberculosis, rifampicin interferes with DNA and protein syntheses by binding to bacterial RNA polymerase (Song et al., 2021). Due to its ability to cross the BBB, rifampicin can exert its antimicrobial action directly in the brain. Rifampicin can also target the Aβ oligomer and promote its dissociation into monomers to inhibit the formation of fibrils (Umeda et al., 2016). In addition, rifampicin can bind to Aβ through hydrophobic interactions between its lipophilic ansa chain and the hydrophobic region of the peptide, thus blocking the linkages between peptide molecules (Tomiyama et al., 1994). Abnormal regulation of permeability glycoprotein (P-gp) may be associated with cognitive impairment in AD, and rifampicin can upregulate P-gp expression (Yulug et al., 2014; Kaur and Sodhi, 2015). Furthermore, rifampicin reduced memory disorders in an AlCl3-treated AD rat model (Kaur and Sodhi, 2015). One milligram of rifampicin per day may improved memory in transgenic mice (Umeda et al., 2016).

A study by Umeda et al. (2016) that administered rifampicin to APPOSK mice (amyloid-β oligomer model) found that treatment reduced Aβ accumulation, provided synaptic protection, and reduced microglial activation. Another study that administered doxycycline and rifampicin observed improvements in cognitive function as assessed by the Standardized AD Assessment Scale–Cognitive Subscale (SADAScog) (Loeb et al., 2004). However, the second study did not find any improvements in cognition or function in patients with mild to moderate AD after doxycycline/rifampicin administration (Molloy et al., 2013). Further investigation is needed to understand why the benefits seen in murine models do not translate to clinical trials.

#### 4.2.3 Anti-*H. pylori*

The current data suggest that *H. pylori* infection may influence the pathophysiology of AD (Doulberis et al., 2018). Amoxicillin is a broad-spectrum antibiotic used to treat a wide range of bacterial infections. In a 2-year study involving 56 histologically *H. pylori*-positive patients with AD, 33 patients received bacterial eradication with triple therapy (omeprazole, clarithromycin, and amoxicillin) and 23 controls did not. *H. pylori* was successfully eradicated in 28 patients with AD (85%). After 2 years, in the subgroup of patients with *H. pylori* eradication, cognitive and functional performance improved significantly but not in other patients (Kountouras et al., 2009).

However, before recommending short-term and cost-effective therapeutic regimens for *H. pylori*-associated AD, further large-scale randomized controlled trials (RCTs) must be conducted to clarify the possible beneficial effects of *H. pylori* eradication on the pathophysiology of AD.

#### 4.2.4 Anti-*P. gingivalis*

Interestingly, bacterial infections mainly associated with periodontal disease have recently been suggested to be involved in the progression of AD. *P. gingivalis*, the main pathogen in chronic periodontitis, has been repeatedly found in the brains of AD patients (Poole et al., 2013; Singhrao et al., 2015), with dysregulated genes in infected macrophages matching those in the hippocampus of AD patients (Carter et al., 2017).

In addition to antibiotics, small molecule inhibitors targeting gingipains, the toxic proteases of *P. gingivalis*, have been developed (Dominy et al., 2019). One of these compounds, COR388, is currently a phase 2/3 clinical trial for AD. In a recent study, COR388 was orally administered to treat older dogs with *Porphyromonas gulae* oral infections and periodontal disease. COR388 inhibited lysine-gingipain and reduced the *P. gulae* load in the saliva, buccal cells, and gingival crevicular fluid (Arastu-Kapur et al., 2020). One study found that COR388 inhibited the growth of *P. gingivalis* in defined growth media. As a narrow-spectrum antibiotic, the lowest dose of COR388 (3 mg/kg) showed a reduction in brain *P. gingivalis* load, but there was no reduction in brain Aβ_1–42_ or TNF-α levels (Dominy et al., 2019). Whether increasing the dose of COR388 is more effective in the treatment of AD requires further clinical verification.

In conclusion, antibiotics can indeed improve pathological changes and cognitive impairment in AD patients. However, the overuse of antibiotics can lead to several side effects, including gastrointestinal disturbances, photosensitivity, and potential impacts on the microbiome, which may result in secondary infections (Panza et al., 2019). The main limitations are the potential for antibiotic resistance and the impact on beneficial microbial flora. Long-term safety and efficacy in human subjects require more extensive research (Panza et al., 2019). Therefore, accurately weighing the benefits and risks of antibiotics can we produce better outcomes for patients with AD.

### 4.3 Anti-inflammatory treatment

Neuroinflammation is an important pathological mechanism that contributes to the pathogenesis of AD. Chronic activation of the immune system leads to the release of pro-inflammatory cytokines and virulence factors (Calsolaro and Edison, 2016). Therefore, anti-inflammatory drugs are also worth considering as potential anti-AD treatments (Gyengesi and Munch, 2020).

Many studies have shown that long-term use of nonsteroidal anti-inflammatory drugs (NSAIDs) has a protective effect in AD patients by reducing or delaying disease progression, although the mechanism of this protective effect remains unclear and there are some conflicting reports on its efficacy (Revesz et al., 2016). Due to the upregulation of cyclooxygenase-1 in the microglia in AD, several epidemiologic studies have shown that NSAIDs reduce behavioral and pathological defects in transgenic mouse models of AD (McGeer and McGeer, 2007). Studies on transgenic mouse models have shown that the use of ibuprofen reduces amyloid plaque deposition, tau phosphorylation in the hippocampal region, and activation of astrocytes and microglia (McGeer and McGeer, 2007; Choi et al., 2013). Epidemiologic and observational studies have shown that NSAIDs may have a protective effect, especially with long-term use. However, in RCTs, NSAIDs did not appear to be effective in the treatment or prevention of disease (Calsolaro and Edison, 2016). A recent Cochrane review of 14 RCTs of aspirin, steroids, and NSAIDs confirmed these results, suggesting that the efficacy of these drugs has not been proven (Jaturapatporn et al., 2012). A meta-analysis showed that the use of NSAIDs was significantly associated with a reduced risk of AD in observational studies; however, NSAIDs did not significantly affect the risk of AD in a single RCT (Wang J. et al., 2015). Therefore, the efficacy of NSAIDs in AD requires further study. Long-term use of NSAIDs can lead to several side effects, including gastrointestinal issues, cardiovascular risks, and renal impairment (Iqbal et al., 2020). Accurately weighing the benefits and risks of NSAID treatment is essential to determine their role in AD therapy.

### 4.4 Fecal microbiota transplantation (FMT) as a therapeutic strategy for AD

Recent research underscores the significance of the gut-brain axis in neurodegenerative diseases, particularly AD, where dysbiosis or microbial imbalance in the gut has been linked to increased inflammation and Aβ deposition, which are the key features of AD pathology (Jin et al., 2023; Zheng et al., 2023; Hao et al., 2024). FMT can restore healthy gut microbiota by transferring fecal matter from a healthy donor to a recipient, which can rebalance gut microbiota and reduce pathogen-induced inflammation. Specifically, FMT modifies the gut microbiota by increasing beneficial bacteria (such as Bacteroidetes) and reducing harmful bacteria (such as *Escherichia*/*Shigella*) implicated in AD inflammation. It lowers the levels of pro-inflammatory cytokines, such as IL-17a, IL-6, and TNF-α, and reduces brain inflammation and microglial activation. FMT inhibits β-secretase activity, enhances gut barrier integrity, and promotes anti-inflammatory cytokines, such as IL-10, thereby decreasing Aβ levels and mitigating chronic inflammation in AD (Hao et al., 2024).

Aβ in the gut may be a significant source of Aβ plaques in the brain, with gut microbiota affecting AD through the gut-brain axis. Gut-derived Aβ42 is primarily transported to the brain via the bloodstream rather than the vagus nerve, and its levels increase with age, particularly in AD model mice (APP/PS1) (Sun et al., 2019). Alterations in the gut microbiota are closely associated with the onset of AD and promote its development by increasing levels of BACE1 and Aβ42 in the gut. FMT from aged APP/PS1 mice to young mice resulted in early AD-like neuroinflammation and cognitive impairment in the recipients, suggesting that FMT may affect AD pathology by modulating gut Aβ production and transport (Jin et al., 2023).

In summary, FMT has the potential to treat AD through multiple mechanisms, including regulation of gut microbiota, reduction in inflammation, and alteration in Aβ production and transport. These findings suggest that FMT could be an effective therapeutic strategy for AD. However, further research is needed to validate its clinical safety and efficacy (Zheng et al., 2023).

## 5 Future research directions

Despite significant advancements in understanding AD pathology’s molecular and cellular mechanisms, many questions remain unanswered. Future research should focus on elucidating the detailed molecular pathways by which pathogens contribute to AD, including the specific interactions between pathogens and key AD proteins such as Aβ and tau. Longitudinal and population-based studies are crucial to identify critical periods when pathogen effects are most significant and uncover potential genetic modifiers of this effect. Therapeutic development should prioritize broad-spectrum antiviral and antibacterial agents that effectively penetrate the CNS. Clinical trials must rigorously test the efficacy of targeted anti-pathogen therapies, ideally in combination with existing AD treatments, to enhance therapeutic outcomes. Identifying reliable biomarkers for the early detection of pathogen involvement in AD is essential for facilitating early intervention and improving patient outcomes. By addressing these areas, future research can significantly advance our understanding of the role of pathogens in AD and pave the way for novel therapeutic strategies.

## 6 Conclusion

Despite significant advances in the study of the molecular and cellular mechanisms of AD pathology over the past few decades, much remains to be done to achieve a comprehensive understanding of the pathogenesis of AD. Mounting evidence suggests that pathogens play a critical role in promoting AD progression. Some related clinical trials have also confirmed the beneficial roles of anti-pathogens in improving pathological changes and cognitive impairment in patients with AD. Owing to the numerous side effects that are associated with anti-infections overuse, only by accurately weighing the benefits and risks of anti-infections can we improve outcomes for AD patients. More pre-clinical and clinical trials are needed in the future to comprehensively evaluate the feasibility of anti-infections for AD.
